# DNA damage in human pterygium: One-shot multiple targets

**Published:** 2013-02-07

**Authors:** Anca Maria Cimpean, Mihai Poenaru Sava, Marius Raica

**Affiliations:** 1 Department of Histology, Angiogenesis Research Center, “Victor Babeş” University of Medicine and Pharmacy Timişoara, Romania; 2 Department of Ophthalmology, „Victor Babeş” University of Medicine and Pharmacy Timişoara, Romania

## Abstract

**Purpose:**

Little is known about DNA damage in human pterygium, and no data about DNA damage involvement as a potential angiogenic factor are available. We studied, with immunohistochemistry, the presence and localization of thymine dimers in the epithelial and stromal components of the human primary pterygium and its recurrences with a special emphasis on the vascular network and its interactions with the p53 tumor suppressor gene protein.

**Methods:**

Thirty-five primary human pterygium, three recurrences, and three normal bulbar conjunctiva were included in the present study. Formalin-fixed, paraffin-embedded tissues were submitted for immunohistochemical analysis with antithymine dimers and p53 antibodies. Thymine dimer and p53 nuclear staining was assessed in the epithelial and stromal components of pterygial tissues and normal counterparts.

**Results:**

Thymine dimers were present in the epithelial and stromal components of human pterygium and its recurrences. The thymine dimers were detected in the epithelial component of the human pterygium with a higher density and intensity in the basal layer of the epithelium. Small blood vessels’ endothelial cells showed positive reaction for antithymine dimer antibodies together with isolated positive expression found in the nuclei of perivascular cells. For the recurrent pterygium, dimer expression was found only in the subepithelial fibrovascular layer components and in scattered cells from the basal layer of the epithelium. P53 expression was positive in 38.5% of the cases in the epithelial compartment, and in two cases, scattered p53 positive endothelial, fibroblast-like, and perivascular cells were detected in the fibrovascular compartment.

**Conclusions:**

Thymine dimers in human pterygium and its recurrences suggest that DNA damage is involved not only in pterygium epithelial and fibrous proliferation but also in angiogenesis and lymphangiogenesis from this ocular lesion in a still incomplete elucidated pathogenic mechanism.

## Introduction

Among pathologic conditions of the eye, human pterygium remains one of the most controversial ocular diseases [1]. Despite its classification by pathologists as a benign lesion, this epithelial and fibrovascular outgrowth of the ocular surface has a proliferative, invasive, and highly vascularized microscopic appearance and acts as a clinically aggressive lesion by invading the cornea and pupillary field [2]. Moreover, the recurrent behavior remains one of the unexplained features of human pterygium. Pterygium etiology includes several factors such as ultraviolet (UV) light damage, dry and dusty environments, and repeated microtrauma together with not fully elucidated immunological mechanisms both humoral (immunoglobulin A [IgA], IgM, and IgG) and cellular (lymphocytes, plasma cells, and mast cells) [3-5].

Ultraviolet light exposure induces the formation of pyrimidine dimers, molecular lesions consisting of thymine or cytosine bases in DNA via photochemical reactions [6]. These lesions have premutagenic features and alter the DNA structure. Thymine dimers may be repaired by photoreactivation, but unrepaired dimers are mutagenic.

Although the role of ultraviolet light in the pathogenesis of human pterygium is widely accepted, data about thymine dimers’ presence and distribution are limited to their description in the normal eye anterior segment from rabbits [7]. Indirect evidence of DNA damage in human pterygium samples has been reported by quantification of oxidative DNA damage [8,9]. No data about thymine dimer expression or potential involvement in the pathogenesis of primary pterygium and its recurrences are available in the literature for human samples.

By regulating normal response to DNA damage, p53 is a key element in maintaining genomic stability. P53 is involved in several aspects of cell cycle arrest, apoptosis, control of genome integrity, and DNA repair [10,11]. UV light exposure induces two types of base alterations (predominant base changes are C→T and CC→TT mutations at dipyrimidine sequences) proved by many experimental systems [12-14] but not certified for human pterygium. Among various types of lesions formed in DNA after UV light exposure, cyclobutane pyrimidine dimers are the most mutagenic lesions based on frequency, slow repair, and distinct mutagenicity [15-17]. The *p53* gene is one of the most sensitive genes for formation of cyclobutane pyrimidine as a mutational hotspot at codon 270 (corresponding to codon 278 in humans) in UV light–induced mouse skin tumors [18,19]. Moreover, it was demonstrated that codon 270 of the *p53* gene had less repair compared with neighboring positions, and this is not the only site at which significant levels of cyclobutane pyrimidine remained and induced mutations in the mouse skin tumor model [20,21]. No similar data have been reported for human pterygium, despite the widely accepted involvement of UV light in its pathogenesis. Starting from these “gaps” concerning basic research on structural and molecular changes following UV light exposure in human pterygium, we proposed to study, with immunohistochemistry, the presence and distribution of thymine dimers and their relationship with p53 protein expression and distribution in the epithelial and fibrovascular compartments of normal conjunctiva, primary pterygium, and its recurrences.

## Material and methods

### Patients and preparation of human tissues

There were recruited 38 patiens. Specimens were collected by surgery from patients (32 men and six women) aged between 28 and 63 years old, admitted to the Ophthalmology Department for visual disturbances because of the encroachment of a fleshy, triangular portion of the bulbar conjunctiva into the cornea in all patients. Normal bulbar conjunctival tissues were obtained from three patients during cataract surgery and were included in this study as control group specimens. In all cases, informed consent was obtained from all individuals enrolled in this study. Research methodology was approved by the Ethics Committee of the Victor Babeş University of Medicine and Pharmacy Timişoara (Romania) and was performed according to the Declaration of Helsinki.

Resected samples were fixed in 10% buffered formalin for 24 h and paraffin embedded by using the automated system protocol (Thermo-Shandon, Loughborough, UK). Five μm serial sections were taken from each paraffin block, and routine hematoxylin and eosin staining was performed for pathologic evaluation.

### Immunohistochemistry

Specimens for the immunohistochemical procedure were chosen with microscopic evaluation of the hematoxylin and eosin–stained slides. Dewaxed sections were then antigen unmasked by using the heat-induced epitope retrieval method (antigen retrieval buffer pH 6, 30 min, 99 °C). After incubation with primary antibody (monoclonal mouse antihuman thymine dimer antibodies [Kamiya Biomedical Company, Seattle, WA, clone KTM53, dilution 1:10,000, and p53 monoclonal antibody, clone DO7 [Dako, Carpinteria, CA], ready to use, 30 min incubation time at room temperature), the specimens were treated with the Novolink polymer detection system (Leica Microsystems, Newcastle, UK). Visualization of the final product was done by using 3,3′-diaminobenzidine, followed by nuclear counterstain with modified Lillie’s hematoxylin (DakoCytomation, Carpinteria, CA). All immunohistochemical procedure steps were performed in an automated manner (by using PT Link, Dako, for the antigen retrieval step and DAKO Autostainer Plus for the next steps).

### Specimen evaluation

Nuclear thymine dimer and p53 expression in human normal conjunctiva, primary pterygium, and its recurrences was quantified with light microscopy (Nikon Eclipse E600 Microscope, Tokyo, Japan) in the epithelial and subepithelial compartments. We evaluated the intensity and density of positive nuclei by counting them on three consecutive microscopic fields at 20× magnification in the epithelial compartment. For the fibrovascular compartment, we observed the nuclear expression in the vascular cells (endothelial and perivascular) and in connective tissue cells with fibroblast and/or myofibroblast morphology.

Statistical analysis was performed by using SPSS software, version 13. Statistical methods included three correlation tests (Spearman, Kendall, and Pearson tests) applied to thymine dimer and p53 expression in the epithelial compartment of human pterygium. A significant correlation was considered for p<0.01.

## Results

### Macroscopic and histopathologic features

**Figure 1:**
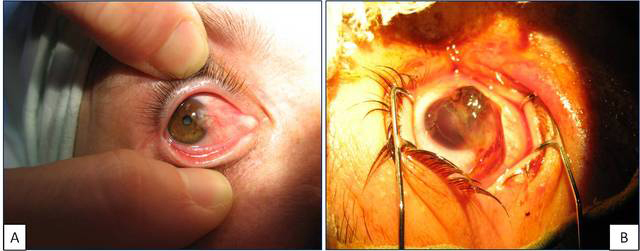
Macroscopic view of primary pterygium (**A**) and recurrent pterygium (**B**).

Human pterygium tissues had varied macroscopic appearance depending on their primary or recurrent status. Often, primary pterygium was composed of a white, fleshy, highly vascularized triangle overgrowth of the nasal portion from the bulbar conjunctiva, with partial visual field invasion (Figure 1A). Recurrent pterygium was huge, invading the entire visual field and having a macroscopic heterogeneous appearance, with pigmented areas mixed with hemorrhagic spots and a visible vascular network (Figure 1B). On hematoxylin and eosin stain, common morphologic features of the epithelial and fibrovascular compartments (goblet cell hyperplasia, increased thickness of the epithelium, subepithelial higher vascularization, fibroelastosis) of the pterygium tissues were observed.

### Thymine dimer expression and distribution

**Figure 2:**
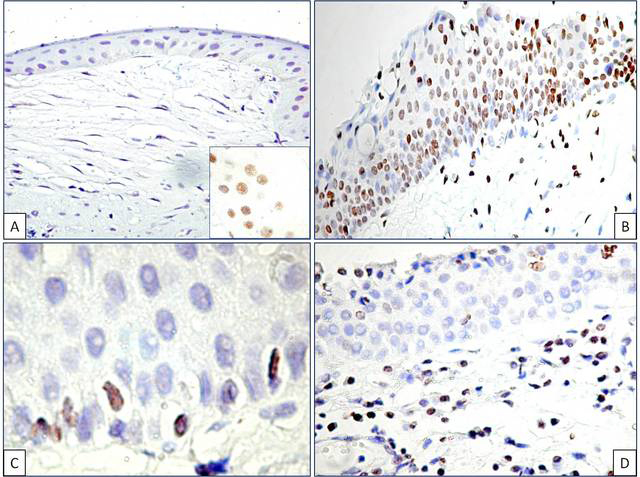
No positive reaction for thymine dimer immunohistochemistry in normal conjunctiva (**A**). Note the weak and inconstant positive reaction in the epithelium but not in the subepithelial layer from one case of normal conjunctiva (a, inset). Nuclear thymine dimer expression in the primary pterygium epithelium (**B**) and recurrent pterygium (**D**). Note the persistence of positive thymine dimer cells in the basal layer (**C**).

Normal conjunctiva epithelial and connective tissue components had no positive reaction to thymine dimer immunostaining in two cases. We observed a weak, inconstant, and focal positivity for thymine dimers in the third specimen of normal conjunctiva; this reaction was restricted to the epithelium (Figure 2A).

Hyperplastic epithelium from primary pterygium had a positive reaction for thymine dimers with relative homogeneous density throughout the entire thickness of the epithelium and heterogeneous intensity. The epithelial and goblet cells had positive nuclei for thymine dimer immunostaining. The density of the thymine dimer positive cells inside the primary pterygium epithelium ranged between 30 and 400/field ×200. The intensity of the thymine dimer immunostaining decreased from the basal through the superficial epithelial layers (Figure 2B). Moreover, clusters of intensely stained nuclei grouped in the epithelial basal layer were identified in primary pterygium tissues. Scattered thymine dimer positive cells were also detected in the basal epithelial layer from recurrent pterygium (Figure 2C). Pterygium epithelium from the recurrent state was characterized by the lack of thymine dimer expression in the suprabasal and superficial layers except isolated positive cells in the most superficial part (Figure 2D).

**Figure 3:**
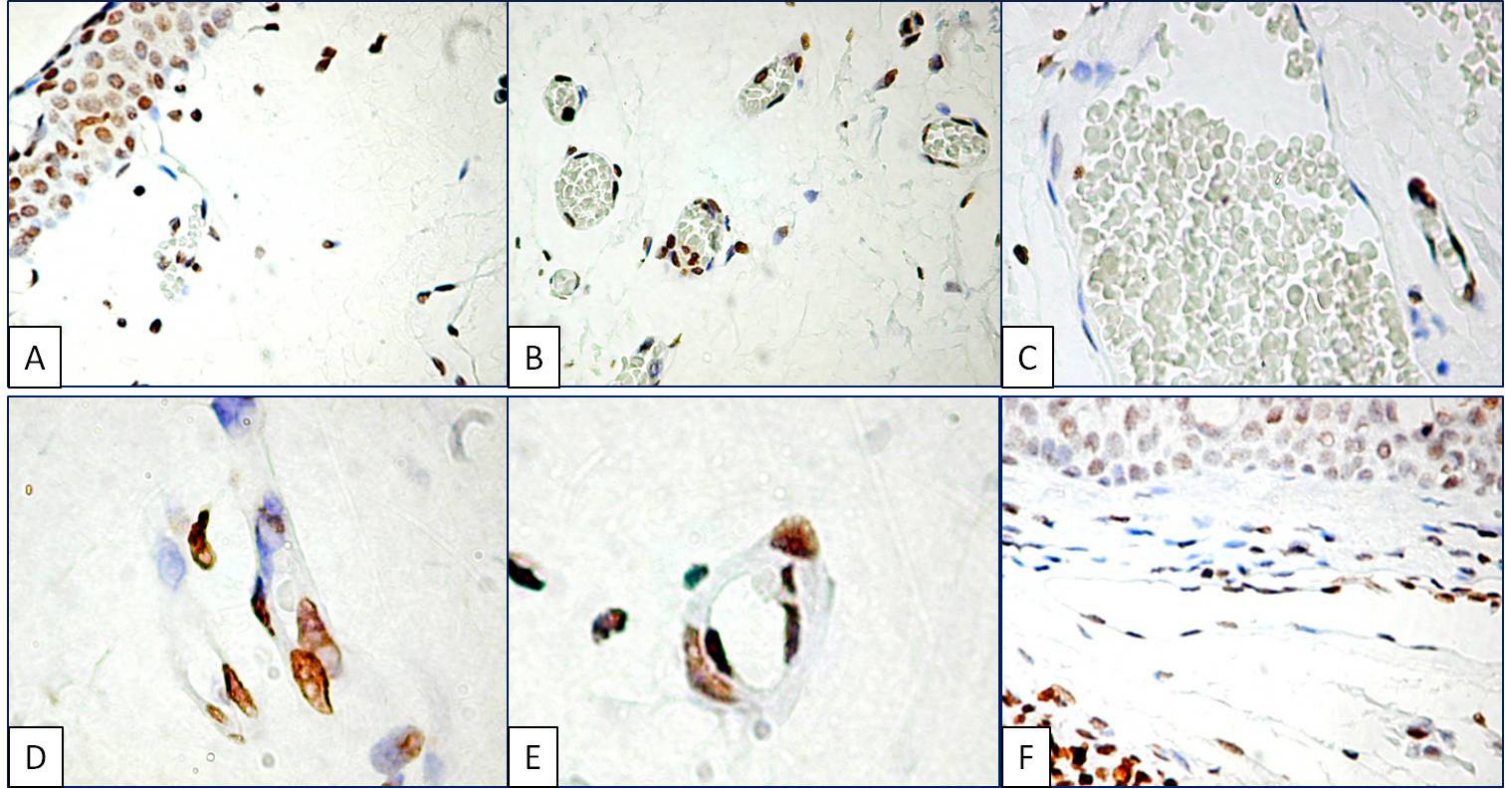
Thymine dimer expression in the fibrovascular compartment of human pterygium. Epithelial and fixed connective tissue cells were positive for thymine dimer reaction (**A**). Small blood vessels’ endothelial cells constantly had nuclear expression in almost all endothelial areas (**B**). In contrast, for mature blood vessels’ endothelial cells from preexisting vessels no immunohistochemical expression of DNA damage was found (**C**). Endothelial cells from the tip of the vascular sprout intensely stained for thymine dimers (**D**). Covering pericytes had a moderate intensity of thymine dimer expression compared with the capillary endothelial cells (**E**). Thymine dimers expressing endothelial cells line the lymphatic vessel lumen (**F**).

As a particular observation, thymine dimer positive nuclei were not restricted to the epithelial layer. Thymine dimer formation was also induced by UV light in the stromal components of the primary and recurrent pterygium (Figure 3A). If the recurrent pterygium epithelial cells lacked thymine dimer expression (as a sign of effective DNA repair), the stromal components from the recurrent state still had a positive reaction for thymine dimers. Connective tissue cells with fibroblast-like appearance expressed thymine dimers in primary and recurrent pterygium (Figure 3B).

The presence of thymine dimers was also detected in the fibrovascular tissue vascular network, in the blood and lymphatic vessels. Endothelial cells from small blood vessels such as capillaries and post-capillary venules but not those from mature vessels intensely expressed thymine dimers in the primary and recurrent status. Positive endothelial cells circumscribed the inner surface of small capillaries and were not visible in the large vessels of the endothelium (Figure 3C). Endothelial cell nuclei from the tip of the vascular sprouts had intense thymine dimer expression (Figure 3D). In addition, a moderate reaction was observed in the pericyte nuclei surrounding the capillaries (Figure 3E). Intense DNA damage in the lymphatic endothelial cells was observed in primary and recurrent pterygium (Figure 3F).

### p53: Friend or foe of human pterygium?

**Figure 4:**
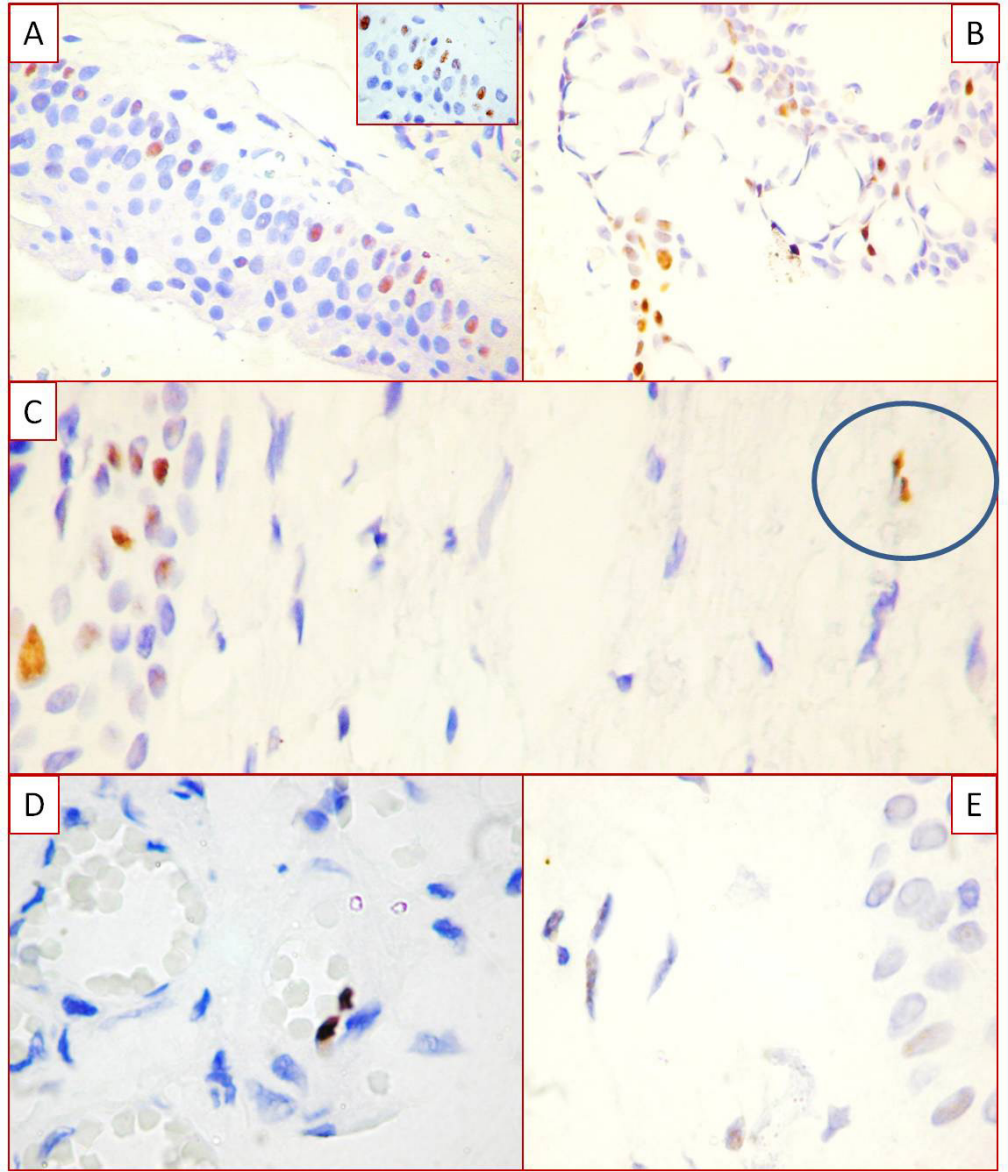
P53 expression in human pterygium. Basal and parabasal distribution of p53 positive cells in the human pterygium (**A**, **B**). Scattered positive p53 positive fibroblast-like cells in fibrovascular compartment of human pterygium (**C**). Note the discrepancies between the higher density of the p53 positive cells in the basal layer of the epithelial compartment and the lack of p53 expression in the stromal component. Blood and lymphatic endothelial cells positive for p53, with high intensity for blood vessels (**D**) and low density for lymphatics (**E**).

**Figure 5:**
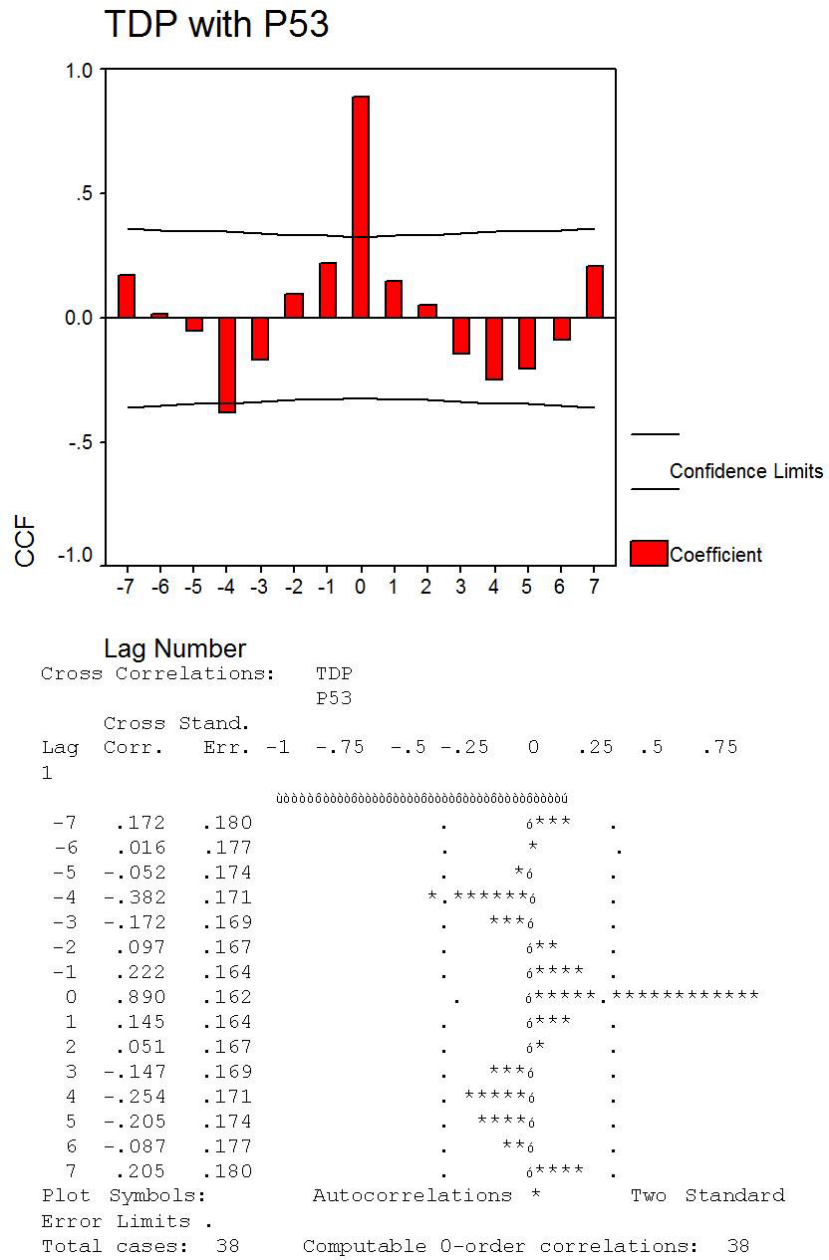
Statistical analysis and cross correlation graphic showing significant correlation between thymine dimer expression in pterygium and p53 presence.

Divergent p53 expression was clearly detected between the epithelial and fibrovascular compartments of normal conjunctiva and primary and recurrent human pterygium. No p53 expression was detected in all three specimens of normal conjunctiva in epithelial or connective tissue compartments. A p53 positive reaction was mostly detected in the epithelial compartment of human pterygium compared with the stromal components. The p53 positivity observed in primary and recurrent pterygium had a specific distribution inside the epithelium, and was positive in the basal and suprabasal epithelial cell nuclei of primary pterygium (Figure 4A,B). Moreover, the highest density of p53 positive nuclei characterized the cases with hyperplastic epithelium where more than 70% of the epithelial cell nuclei were positive for thymine dimers. A significant correlation was found between the number of thymine dimers and p53 positive nuclei in human pterygium epithelial cells (p<0.001, Figure 5). In the fibrovascular compartment, p53 expression was found only in two cases and detected in the fibroblast-like cells (Figure 4C) and in the endothelial cells of blood and lymphatic vessels (Figure 4D,E). No differences between primary and recurrent pterygium were detected concerning p53 presence and distribution.

## Discussion

An accurate description of human primary and recurrent pterygium histopathology was previously performed by Chui et al. in 2011 [22]. The authors launched the hypothesis that primitive cell clusters of basal epithelial cells are responsible for the invasive behavior and recurrences of human pterygium through their activation as a result of UV radiation. Using thymine dimer immunostaining, we observed clusters of intensely stained positive thymine dimer cells grouping in the basal portion of the epithelium from primary pterygium. Moreover, for recurrent pterygium, only scattered basal cells retained positivity for thymine dimers as a sign of a possible lack of the DNA repair mechanism. It is already recognized that the defects in the DNA repair mechanism can cause genetic instability, increased and uncontrolled proliferation, and irreversible errors in protein sequences [8]. The persistence of thymine dimer positive basal cells from the recurrent pterygium specimens observed in the present study for recurrent pterygium could explain, in part, the cellular origin of recurrences in this benign eye lesion by possible mutations due to an ineffective DNA repair mechanism.

Based on our observation concerning the presence of thymine dimers in the fibrovascular compartments, some aspects regarding fibroblasts and endothelial and perivascular cells must be discussed. All of these cells expressed thymine dimers in the primary pterygium specimens. Our results revealed, for the first time, divergent thymine dimer expression in the epithelial compartment (negative for this marker) and the fibrovascular compartment (positive) in the recurrent pterygium. Human pterygium fibroblasts are the most studied and controversial cells from this eye lesion [23-25]. Indirect evidence of DNA damage by an oxidative mechanism had been reported for human pterygium evaluated exclusively by the formation of 8-hydroxydeoxyguanosine (8-OhdG), but positivity for this marker was inconstant in less than 23% of the human pterygium tissues [8,26,27]. The cyclobutane pyrimidine dimers group (which includes thymine dimers) has not been studied on human pterygium specimens until now. Only one study reported the formation of ultraviolet light-induced cyclobutane pyrimidine dimers in rabbit corneas in an experimental study [7]. The persistence of thymine dimer expression in the recurrent pterygium observed in our study suggested the failure of DNA repair in human pterygium fibroblasts. This unrepaired DNA damage in the fibrovascular compartment may responsible for the UV light–induced gene mutations and polymorphisms recently extensively reported in human pterygium [26-30]. Consecutively, this can induce recurrent behavior by the human pterygium.

Angiogenesis and lymphangiogenesis are widely accepted but incomplete characterized processes involved in the pathogenesis of human pterygium. Several mechanisms for blood and lymphatic vessel development in human pterygium have been proposed, including inflammation, growth factor overexpression, hypoxia, and transcription factors [31-36]. UV light–induced angiogenesis was reported many years ago by Bielenberg et al. [37], who demonstrated increased endothelial cell proliferation (bromodeoxyuridine + CD31 + cells) within existing blood vessels, leading to telangiectasia and new blood vessel development in mice skin due to a UV radiation–induced imbalance between a positive angiogenic molecule (basic fibroblast growth factor) and a negative angiogenic molecule (interferon-beta). Recently, a research team from the Shiseido Research Center, Japan, reported UVB-induced alterations to the blood and lymphatic systems in skin [38,39] and launched potential strategies to block photoaging of the skin by inhibiting angiogenesis and/or promoting lymphatic vascular function. The present study reported UV light–induced DNA damage in endothelial cells from human pterygium blood vessels and suggested the involvement of UV light–induced angiogenesis in the pathogenesis of the human primary and recurrent pterygium as a potential therapeutic target.

If UV light–induced DNA damage has already been recognized in the pathogenesis of the skin lesions and now described in human pterygium, no data are available about DNA damage in the pericytes. We highlighted here, with immunohistochemistry, the presence of the thymine dimer positive reaction in the pericyte nuclei surrounding small blood vessels from the fibrovascular compartment of primary and recurrent pterygium. The potential role of the consequences of pericyte DNA damage on the angiogenic process and its involvement in other human pterygium pathogenesis steps are completely unknown at this moment.

P53 is involved in mechanisms of anticancer function, and plays a role in apoptosis, genomic stability, and inhibition of angiogenesis. The role of p53 in UV light–induced DNA damage and repair was certified by several studies conducted on UV light–induced skin carcinogenesis or by UV irradiation of cultured lung, breast, or colon cell lines. Two years ago, Smith and Kumar discussed the two faces of tumor suppressor p53 [40]. After UV light exposure, p53 serine residues become phosphorylated and induce DNA repair. This process depends on the UV wavelength and the dose of UV radiation [41,42]. The lesion frequency (DNA damage) promotes p53 “activation” (defined as transcriptional induction of p53-regulated genes) [40]. These data are correlated with our findings. We observed that the highest number of p53 positive cells were found inside the epithelial compartment of human pterygium where more than 70% from total epithelial cells were positive for thymine dimers as a sign of strong DNA damage. Persistence of p53 positivity in the suprabasal and basal layer cells observed in 38.5% of our cases suggested that these cells are preferentially sensitive to UV radiation and might be p53 mutant or p53-null cells as other authors previously reported for other cell types or by using experimental models [41-44]. A p53 mutant background may be mutagenic, and therefore, additional mutations throughout the genome would accumulate in the p53-mutant cells. This is in concordance with previous studies that suggested that basal cells of the human pterygium epithelium are highly unstable and can acquire mutations [22,45]. These mutations could promote cell survival and might be responsible for recurrences of human pterygium.

Two major pathways are recognized as involved in p53-dependent DNA repair in cells exposed to UV radiation: nucleotide excision repair and base excision repair (BER) [29]. Wild-type p53 cells carry out normal BER while mutant p53 cells or null for p53 are defective in BER [46]. Based on these previous findings, persistence of thymine dimer expression together with the absence of p53 expression in the stromal components of most of our cases suggested the lack of a p53 protective role against UV light induces DNA damage in the endothelial cells and fibroblast cells from the fibrovascular compartment of human pterygium. Previously reported findings about the persistence of cyclobutane pyrimidine at codon 270 of the *p53* gene with less repair compared with the neighboring position demonstrated cell mutations in mouse skin tumor models [20]. We speculate, based on our results, that a similar mechanism could be involved in thymine dimer persistence followed by induction of mutational changes in the stromal cells of human pterygium. These mutations from epithelial and stromal cells could be responsible for the higher number of recurrences and resistance to conventional therapies. Further molecular and genetic studies are needed to elucidate the involvement of the *p53* gene in thymine dimer repair from human pterygium.
